# Complaints of the arm, neck and shoulder among computer office workers in Sudan: a prevalence study with validation of an Arabic risk factors questionnaire

**DOI:** 10.1186/1476-069X-7-33

**Published:** 2008-06-27

**Authors:** Shahla M Eltayeb, J Bart Staal, Amar A Hassan, Salwa S Awad, Rob A de Bie

**Affiliations:** 1 Department of Epidemiology and Caphri Research School, Maastricht University, PO Box 616 - 6200 MD Maastricht, The Netherlands; 2 School of Psychology, Ahfad University for Women, PO Box 167, Omdurman, Sudan; 3 School of Health Sciences, department of physiotherapy, Ahfad University for Women, PO Box 167, Omdurman, Sudan

## Abstract

**Background:**

Complaints of the arm, neck and/or shoulders (CANS) in general and computer-related disorders in particular affect millions of computer office workers in Western developed countries. However, with the widespread use of computer systems in developing countries, the associated musculoskeletal complaints are yet to be investigated.

**Aim:**

To study the prevalence of work-related CANS, among computer office workers in Sudan, and to test the psychometric properties of a translated Dutch questionnaire in Arabic language.

**Methods:**

In 2005 282 computer office workers at a mobile telecommunication company and three banks in Khartoum, Sudan, received an Arabic language version of the validated Maastricht upper extremity questionnaire (MUEQ). The questionnaire holds 109 items covering demographic characteristics, in addition to six main domains (i.e. work station, body posture, break time, job control, job demands and social support) assessing potential physical and psychosocial risk factors. Forward/backward translation of the MUQE was done independently by two different translators. Prevalence over the past year were computed for CANS. Further, the psychometric properties of the Arabic questionnaire were investigated (i.e. factor structure and reliability) and cross-validation was carried out.

**Results:**

The response rate of the questionnaire was 88% (n = 250). The one-year prevalence of CANS showed that 53% of the respondents could be classified as mild cases. The highest incidences were found for neck and shoulder symptoms (64% and 41% respectively). The analysis of the psychometric properties of the scale resulted in the identification of 2 factors for each of the 6 domains (i.e. office equipment, computer position, head and body posture, awkward body posture, autonomy, quality of break time, skill discretion, decision authority, time pressure, task complexity, social support, and work flow). The calculation of internal consistency and cross validation provided evidence of reliability and lack of redundancy of items.

**Conclusion:**

The prevalence of CANS among the targeted population seems to correspond strongly with prevalence of CANS in Western developed countries. The Arabic translation of the MUEQ has satisfactory psychometric properties to be used to assess work-related risk factors for the development of CANS among computer office workers in Sudan.

## Background

Complaints of the arm, neck and/or shoulder (CANS) are defined as "musculoskeletal complaints of arm, neck and/or shoulder not caused by acute trauma or by any systemic disease"[1]. CANS affect millions of computer office workers in Western developed countries [2]. However, with the wide use of computer systems in the developing countries [3], the associated musculoskeletal complaints are yet to be investigated.

CANS are the leading cause of occupational illness in the United States with related absenteeism and medical expenses costing the industry between $45 to $54 billion annually [4]. In the Netherlands, with a working population of 7 million, annual costs for these musculoskeletal disorders are estimated to be 2.1 billion Euro [4]. However, very limited data is available about the magnitude of this problem in non-Western regions such as Africa [3], and none so far documented the extent of the problem in Sudan.

In general, the clinical, epidemiological and social aspects of CANS remain largely controversial in the medical literature. According to several reviews, positive but no conclusive relations have been found between various physical and psychosocial risk factors and the occurrence of CANS such as awkward body postures, repetitive movements and psychosocial job characteristics such as high job demands, having low job control and low social support [2,4,5]. The relationships reported in the literature are often derived from cross-sectional studies and mostly from studies carried out in Western countries. In order to investigate causal relations between both physical and psychosocial risk factors and CANS further prospective cohort studies are needed [5]. An example of such a study is the NUDATA study among Danish computer workers, which showed that mouse and keyboard use were associated with an increased risk of carpal tunnel syndrome, elbow and wrist/hand symptoms, forearm pain, and neck and shoulder symptoms [6-10].

The present study aims to translate and validate the Dutch musculoskeletal upper extremity questionnaire (MUEQ), which can be used to assess the occurrence, nature and several work-related physical and psychological risk factors for the development of CANS in the targeted population The second aim of this study is to assess the prevalence of CANS in a Sudanese working population The psychometric properties of the Dutch version of this questionnaire have already been reported in another paper [11]. The psychometric properties of the Arabic translation are reported in the present paper.

## Methods

### Study population and data collection

We conducted a cross-sectional study between April and May 2005. The study population consisted of 282 workers who were invited to participate in the study at two different work locations (Telecommunication Company and three banks) in Khartoum, Sudan.

In order to be included, office workers had to perform jobs with a variety of (1) computer tasks (i.e. administrative, graphical and data entry tasks), and (2) they had to have been employed in the current position for at least six months. Participants were excluded on the basis of the following criteria: (1) severe psychiatric or behavioral disorders (requiring treatment in the last 30 days); (2) having had previous surgery of the upper extremity.

The targeted company and the three banks represent both private and governmental sectors. They all share similar working conditions: i.e. working on average eight hours per day; six days per week in the banks and five days per week in the telecommunication company and they share the same labour legislation. This means that they have the right of a three month fully paid sick leave after which payment declines by half every three months for a maximum period of one year. In the period that follows the employee receives a disability pension of about one third of the original salary (according to the Sudanese labour law of 1997). The selected work locations are situated in modern office buildings and the offices have state-of-the-art lighting, air-conditioning and work stations.

Data were collected with self-administered questionnaires. On the first of April 2005 the questionnaires were distributed among the participants by handing them out at the workplace. Participants were asked to fill out the questionnaire and return it using specially provided boxes. By mid April a reminder note was posted to non-responders, and the end of April 2005 was set as the latest return date. Completed and returned questionnaires were coded and entered in the SPSS 11.0 software program and data were cleaned and made ready for statistical analysis.

We obtained ethical approval of the Ahfad University medical ethical review board, Sudan, for the data collection.

### The questionnaire

Items included in the questionnaire were taken from the MUEQ which was developed in 1999. The psychometric properties of the Dutch version of this questionnaire have been investigated and were found to be valid and reliable [11]. The MUEQ is a screening instrument that allows assessment of the prevalence of CANS and risk factors for the development of these complaints

The MUEQ was translated into Arabic language (the standard written Arabic in the Arab world) with a forward and backward translation procedure. Two bilingual translators (Dutch-Arabic) independently translated the original scale once. They were encouraged to strive for idiomatic rather than word-for-word translation. The Arabic version was then reviewed by several Sudanese experts consisting of an orthopaedic surgeon, psychologist, one physiotherapist, a statistician and an Arabic linguist to assess the necessity of performing a cultural adaptation and to fine-tune it for use among Sudanese workers. A backward translation of the reviewed version was then translated into Dutch, to verify that the meaning of each item of the scale was preserved.

The Arabic version consists of six pages with 109 items and has a completion time of approximately 30 minutes. The Arabic questionnaire covers demographical information of the subjects under study in addition to six main domains as in the MUEQ. These were the following domains: (1) work station; (2) body posture; (3) break time; (4) job control; (5) job demands, and (6) social support. A couple of items assessed the worker's work environment and the frequency and nature of upper extremity complaints (i.e. the presence of complaints in the neck, shoulder, upper and lower arm, elbow, hand and wrist). Further items specified the clinical manifestations of the complaint (i.e. tingling, numbness, weakness, swelling, stiffness, fatigue, continuous pain and change in skin colour or temperature). All items were rephrased as statements in either a five point scale (completely true-completely false) and (always-never) or a dichotomous statement (yes-no). A body mannequin was added to the Arabic version to illustrate the upper extremity anatomical areas. The Arabic questionnaire is presented in Appendix 1. The original Dutch questionnaire and a translated English version have been presented in a separate paper [11].

### Calculation of the prevalence

The prevalence of complaints over the past twelve months lasting for at least one week were computed including 95% confidence intervals for each upper musculoskeletal body region (neck, shoulder, arm, elbow, hand and wrist).

Further, participants who reported complaints in the upper extremity were classified into two groups: (1) mild cases: subjects who reported pain or/and complaints in one or more of the body regions neck, shoulder, hand, wrist and elbows for at least seven days during the preceding 12 months; (2) severe cases: subjects who reported pain or/and complaints in one or more of the body regions neck, shoulder, hand, wrist and elbows for at least seven days during the preceding 12 months while the pain was chronic and present even after a short rest. The prevalence of complaints for mild and severe cases for the past twelve months was computed for males and females including 95% CI. A p-value ≤ 0.05 was considered statistically significant.

In order to investigate to what extent symptoms were spread over the upper extremity prevalence including 95% CI were calculated for the following combinations of body regions: (1) Neck, shoulder, upper arm, elbow, lower arm, hand and wrist symptoms; (2) Neck, shoulder and upper arm symptoms, and (3) Neck and shoulder symptoms.

### Factor analysis

Exploratory factor is a technique used to analyse interrelations among a large number of items (questions) while trying to explain these items in terms of their common underlying dimensions [12]. The purpose of the factor analysis was to divide the items for each of the six domains into at most two factors. We conducted Principal Component Analysis (PCA) with Varimax rotation. The number of factors retained was derived by considering the magnitude of the eigenvalues, Kaiser's (1960) eigenvalues [greater than 1] rule, the proportion of variance extracted, item content, and the interpretability of the resulting factors. Independent factors were considered as meaningful when they appeared before the break in the Scree plot results. As for factor loading after the Varimax rotation, items with a factor loading less then 0.5 on all factors were excluded, unless they represent an essential assumption. Further, each factor had to comprise at least three items. If the results indicated more than two factors, a forced two factor analysis was performed.

### Reliability and internal consistency of the questionnaire

We investigated the internal consistency by calculating Cronbach's alpha and by calculating item-total correlations for each factor that was identified with the factors analysis. An alpha greater than 0.70 was considered acceptable and optimal item-total correlation was considered to be between 0.2 and 0.5 [12].

### Performance of cross-validation

In order to test the stability of the factor structure cross-validation was carried out. Cross-validation, is the **statistical **method of **partitioning **a **sample **of **data **into subsets such that the analysis is initially performed on a single subset, while the other subset(s) are retained for subsequent use in confirming and validating the initial analysis [13]. The initial subset is called the training set; the other subset(s) are called validation or testing sets. The sub-sample (n = 125) was randomly selected from the study population.

## Results

### Demographic characteristics of the study population

Two hundred and fifty men and women out of the 282 responded to the baseline questionnaire which resulted in a response rate of 88%. Sixty-five percent (n = 163) were men. Eighty percent of the total sample was aged between 25 and 35 years, 76% of the males and 87% of the females were also in this age group. Of the female participants, 65% worked 6 to 8 hours per day with a computer compared to 59% of the male participants and 60% of the entire study population. Fifty-eight percent of the females had worked between 2 and 4 years in their current position compared to 47% of the males (Table 1).

**Table 1:** Descriptive characteristics of the study population*

	**Male N = 163**	**Female N = 83**
Gender	65.2%	34.8%

Age		
25–35	76.1	87.4
36–45	17.8	12.6
46–55	6.1	0.0

Numbers working hours/Day		
4 to7 hrs	37.4	46.0
8 hrs	48.5	48.3
More than 8 hrs	14.1	04.6

Numbers of working hours with computer/Day		
3 to 5 hrs	31.9	27.6
6 to 8 hrs	59.5	65.5
> 8 hrs	08.0	02.3

Numbers of working years in current position		
6 month to 1 year	28.8	26.4
2 to 4 years	47.2	58.6
5 years and more	23.9	14.9

*Total Number of Subjects = 250

### Prevalence of CANS

The 12-month complaints prevalence including 95% CI confidence intervals are presented in table 2. The most commonly reported complaints were neck and shoulder symptoms (64% and 41% respectively), followed by upper arm, hand and wrist complaints (32%, 30% and 29%) and lower arm and elbow complaints (21% and 19%). Fifty-three percent of the respondents were mild cases, of whom 51% were male. The total percentage of severe cases was 9% of whom 66% were females.

**Table 2:** Prevalence of CANS during the previous year that lasted at least one week

**Complaint**	**Total number of ** **subjects with** **complaints**	**Total ** Prevalence (95% CI) (n = 250)	**Male ** Prevalence (95% CI) (n = 163)	**Female ** Prevalence (95% CI) (n = 87)
Neck complaints	161	0.64(0.58 to 0.70)	0.65 (0.57 to 0.72)	0.63 (0.52 to 0.73)
Shoulder complaints	103	0.41 (0.35 to 0.47)	0.37 (0.29 to 0.44)	0.48 (0.37 to 0.58)
Upper arm complaints	82	0.32 (0.26 to 0.38)	0.26 (0.19 to 0.33)	0.44 (0.34 to 0.55)
Elbow complaints	48	0.19 (0.14 to 0.24)	0.16(0.10 to 0.22)	0.24 (0.14 to 0.33)
Lower arm complaints	53	0.21 (0.16 to 0.26)	0.19 (0.12 to 0.25)	0.25 (0.15 to 0.34)
Wrist complaints	74	0.29 (0.23 to 0.35)	0.24(0.17 to 0.31)	0.43(0.33 to 0.54)
Hand complaints	77	0.30 (0.25 to 0.36)	0.23 (0.17 to 0.30)	0.39 (0.28 to 0.49)
Mild cases	133	0.53 (0.48 to 0.60)	0.51 (0.42 to 0.59)	0.58 (0.47 to 0.68)
Severe cases	9	0.04 (0.07 to 0.17)	0.33 (0.30 to 0.47)	0.66 (0.53 to 0.74)

**Figure 1:**
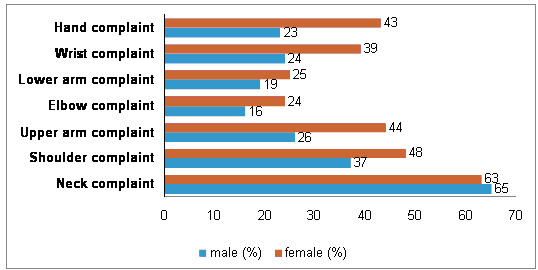
Percentage of upper extremity musculoskeletal complaints during the previous year that lasted at least one week for males and females.

The proportion of participants with complaints of the various upper extremity body regions (except for neck pain and low arm pain) was greater for females than for males (Table 2 and figure 1). This difference was statistically significant for the hand and upper arm regions. The distribution of the complaints by anatomical localization (i.e. left side, right side and both sides) classified by gender is presented in table 3. The results indicated that in general "right side" complaints were reported more frequently than "left side" complaints.

**Table 3:** Percentage of CANS during the previous year, enduring one week distributed by locality

**Percentages**	**Complaint anatomical area**					
**Male ** **(N = 163)**	Shoulder complaints	Upper arm complaints	Elbow complaints	Lower arm complaints	Wrist complaints	Hand complaints

Right side	10.4	14.1	05.5	08.6	16.6	16.6
Left side	02.5	01.8	02.5	01.8	01.8	01.8
Both sides	24.5	10.4	08.6	08.6	05.5	06.1

**Female ** **(N = 87)**	Shoulder complaints	Upper arm complaints	Elbow complaints	Lower arm complaints	Wrist complaints	Hand complaints
Right side	17.2	26.4	17.2	14.9	32.2	28.7
Left side	04.6	01.1	06.9	0.0	0.0	0.0
Both sides	26.4	17.2	06.9	10.3	11.5	10.3

Nine percent of the entire sample reported complaints of the entire upper extremity, whereas 19% reported complaints of the neck, shoulder and upper arm and 41% of neck and shoulder complaints (table 4).

**Table 4:** Prevalence of CANS in combinations of body regions during the previous year enduring one week

**Complaints**	**Total number of subjects** ** with complaints**	**Total ** Prevalence (95% CI) (n = 250)
Neck, shoulder, upper arm, elbow, lower arm, hand and wrist symptoms	24	0.09 (0.12 – 0.05)
Neck, shoulder and upper arm symptoms	49	0.19 (0.24 – 0.14)
Neck and shoulder symptoms	104	0.41 (0.47 – 0.34)

### Psychometric characteristics of the questionnaire

Because the social and environmental conditions in Sudan vary from those in Western European and North American countries, a simple translation was not suitable. Thus, the investigators added some new questions. Seven items were added (item nr. 56, 59, 71, 74, 75, 76 and 106; see appendix 1) to be in keeping with the Sudanese work setting, environment and to tackle some aspects that are more likely to be performed by the Sudanese workers, for example having a breakfast break at the office.

Results from the factor analysis indicated that each domain included two factors accounting for approximately 40% of the variance. Cronbach's alpha coefficients for the majority of the factors in the questionnaire were greater than the accepted number of 0.70 [12]. However, some of the factors (i.e. computer position, task complexity, and autonomy and office equipment) showed an alpha below 0.65 and showed suboptimal item-total correlation (either below 0.2 or above 0.5).

### Results of the cross-validation

We found that the number of factors, the factor structure and factors loadings were for the greater part comparable between the first randomly created sub-sample and the total sample. Differences were found in the 'quality of work break' domain. The items "I can divide my work tasks", "I find my work breaks sufficient", "I can decide when to take a break" and "I can decide when to start and to stop" load positively on the first factor in the randomly selected sub-sample; however, the same items load highly on the second factor in the total sample analysis. No important differences were further found between the results of the total sample analysis and the randomly selected sub-sample. We therefore only present the results of the factor analyses as applied to the randomly created first sub-sample (table 5). The results of the factor analyses are presented in table 5. The results of the internal consistency analyses and item-total correlations are presented in table 6 and 7 respectively.

**Table 5:** Factor loadings identified using principal component analysis and the orthogonal VARIMAX rotation *

**Domain**	**Abbreviated item description**	**Factor1**	**Factor2**
**Work Station**		Office equipment	Computer position

	My desk at work has suitable height	0.54	0.22
	I have enough space to work at my office	0.51	0.42
	I have a file holder I use when I am typing	0.74	0.04
	My chair supports my lower back	0.57	0.30
	Keyboard is placed directly in front	0.42	0.69
	I sit straight in front of screen	0.03	0.68
Eigenvalue		1.72	1.42
% of Variance		21.5	17.9

**Body Posture**		Head and body posture	Awkward body posture

	I find my job physically exhausting	0.98	0.01
	When I work my hand is placed in a straight line	0.55	0.01
	When I work my head is bended	0.98	0.03
	When I work my head is twisted towards the left or right	0.98	-0.01
	When I work my body is twisted towards the left or right	0.98	-0.06
	I sit in a symmetrical position	0.97	-0.04
	I sit for long hours in one position	-0.06	0.87
	For 2 hours per day I sit with lifted shoulders	0.05	0.88
	During my work I sit in an awkward posture	0.02	0.98
	My work requires performing repetitive tasks	0.07	0.92
Eigenvalue		7.10	1.55
% of Variance		24.0%	17.5%

**Break Time**		Autonomy	Break quality

	I can divide my work tasks	0.67	0.47
	I find my work breaks sufficient	0.68	0.28
	I can decide when to take a break	0.77	0.08
	I can decide when to start and to stop	0.78	0.21
	I alternate in my body position	0.06	0.59
	I alternate in my job task	0.41	0.65
	I perform job tasks without a computer	0.53	0.59
	After 2 hours work I take a break for at least 10 minutes	0.31	0.51
	My breaks are spent outside the office	0.36	0.79
Eigenvalue		3.41	1.65
% of Variance		37.9%	18.4%

**Job Control**		Skill discretion	Decision authority

	I participate with other colleges in decision making	0.75	0.64
	I participate in implementation of job tasks	0.75	0.64
	My work develops my abilities	0.72	0.67
	In my work I have the chance to learn new things	0.74	0.65
	I have to be creative in my work	0.73	0.66
	I undertake different tasks in my work	0.77	0.62
	I decide how to perform my job task	0.66	0.73
	I determine the time & speed job tasks	0.63	0.76
	I solve work problems by my self	0.64	0.75
Eigenvalue		8.83	0.06
% of Variance		48.7	12%

**Job Demands**		Time pressure	Task complexity

	I find trouble to finish my job tasks	0.76	0.21
	I take regular over times	0.80	-0.02
	I have limited time to finish my job	0.74	0.13
	I work with max speed to finish my tasks	-0.19	0.59
	I work under extensive work pressure	0.14	0.73
	I find my work tasks difficult	0.40	0.62
Eigenvalue		2.36	1.12
% of Variance		33.7	16.1

**Social Support**		Social support	Work flow

	I find support from supervisors	0.92	0.33
	I receive positive comments	0.93	0.33
	My colleagues are helpful	0.81	0.26
	My supervisors are helpful	0.93	0.33
	I get personal advice from my colleagues	0.92	0.33
	My supervisors are considerate	0.81	0.25
	No contacts with other colleagues	0.92	0.32
	The work flow goes smoothly	0.47	0.80
	I can ask and enquire in my work	0.47	0.81
	My work tasks depend on other colleagues	0.18	0.90
	The work atmosphere is comfortable	0.18	0.90
	I find support from colleages	0.47	0.81
Eigenvalue		8.96	1.72
% of Variance		53.2%	35.8%

Only items with a factor loading >0.5 on at least one factor are reported

**Table 6:** Internal consistency of the twelve Factors

**Domain**	**Factors**	**Internal consistency ** **(Cronbach's alpha)**	**Items numbers**
Work Station	Factor 1: Office equipment	0.50	13.17.18.20
	Factor 2: Computer position	0.48	16.19
Body Posture	Factor 3: Head and body posture	0.88	25.26.27.28. 29.30
	Factor 4: Awkward body posture	0.66	21. 22. 24. 32
Break Time	Factor 5: Autonomy	0.76	50.51.54.55
	Factor 6: Break quality	0.79	47.48.52.53.56
Job Control	Factor 7: Skill discretion	0.84	31.32.33.34.36.37
	Factor 8: Decision authority	0.76	35.38.39
Job Demands	Factor 9: Time pressure	0.71	41.42.45.
	Factor 10: Task complexity	0.53	40.43.46
Social Support	Factor 11: Social support	0.94	70.71.72.73.74.75.76
	Factor 12: Work flow	0.76	65.66.67.68.69

**Table 7:** Item-total correlation of the twelve Factors

**Domain**	**Factors**	**Item-total correlation ** **(Min-Max)**	**Items numbers**
Work Station	Factor 1: Office equipment	0.23 to 0.35	13.17.18.20
	Factor 2: Computer position	0.36	16.19
Body Posture	Factor 3: Head and body posture	0.57 to 0.90	25.26.27.28. 29.30
	Factor 4: Awkward body posture	0.40 to 0.96	21. 22. 24. 32
Break Time	Factor 5: Autonomy	0.23 to 0.47	50.51.54.55
	Factor 6: Break quality	0.43 to 0.62	47.48.52.53.56
Job Control	Factor 7: Skill discretion	0.41 to 0.72	31.32.33.34.36
	Factor 8: Decision authority	0.44 to 0.53	35.38.39
Job Demands	Factor 9: Time pressure	0.12 to 0.30	41.42.45.
	Factor 10: Task complexity	0.49 to 0.56	40.43.46
Social Support	Factor 11:Social support	0.85 to 0.88	70.71.72.73.74.75.76
	Factor 12: Work flow	0.71 to 0.97	65.66.67.68.69

#### Work station

The first group of items addressed the work station (i.e. table, chair and computer placement) and consisted of eight items. Three factors were extracted (data not shown), therefore, we undertook a forced two-factor solution. Examination of the factor loadings showed that two items ("I can adjust my chair height" and "when I use the mouse device my hand is straight") load poorly (>0.5) on both factors. They were therefore excluded. The first factor held four items ("my desk (table) at work has suitable height", "I have enough space to work at my office", "I have a file holder I use when I am typing" and "My chair supports my lower back"). This first factor, which was related to office equipment, accounted for 21.5% of the total variance and had a low Cronbach's alpha of 0.50 while values of item-total correlations varied between 0.23 and 0.35. The second factor included two items ("my keyboard is placed directly in front of me" and "I can sit straight in front of the computer screen"). They were related to the computer position and accounted for 17.9% of the total variance. This factor holds less than three items had a low Cronbach's alpha 0.48 and the item-total correlation was 0.36.

#### Body Posture

The second domain addressed body posture and consisted of 11 items. Two factors were extracted. The Scree plot and the examination of the rotated factor loadings showed that one item ("during my work I use a foot support") load poorly on both factors justifying deletion of this item. The first factor, included six items related to head and body posture ("I find my job physically exhausting", "When I work my hand is placed in a straight line", "When I work my head is bended", "When I work my head is twisted towards the left or right side", "When I work my body is twisted towards the left or right side" and "I sit in a symmetrical position") accounting for 24.0% of the total variance, with a Cronbach's alpha of 0.88, and item-total correlations ranging from 0.57 to 0.90.

The second factor included four items related to an awkward body posture ("during my work I sit for long hours in one position", "for more than two hours per day I work with lifted shoulders", "during my work I sit in an awkward posture" and "my work requires performing repetitive tasks") accounting for 17.5% of the total variance. Cronbach's alpha was 0.66 and the item-total correlations ranged from 0.40 to 0.96.

#### Break Time

Break time during working hours was investigated by 9 items. The Scree plot results identified two factors and examination of the rotated factor loadings showed that the first factor holds four items ("I can divide my work tasks", I find my work breaks sufficient", "I can decide when to take a break" and "I can decide when to start and stop") which made the autonomy scale accounting for 37.9% of the total variance. Cronbach's alpha was 0.76 and the item-total correlations of the autonomy factor ranged from 0.23 to 0.47. Five items related to break quality load highly on the second factor ("I alternate in my body position", "I alternate in my job task", "I perform job tasks without a computer", and "after two hours work I take a break for at least 10 minutes") accounting for 18.4% of the total variance, with a Cronbach's alpha of 0.79 and item-total correlations ranging from 0.43 to 0.62.

#### Job Control

The job control domain included 9 items. The Scree plot results identified two factors. The rotated factor loadings indicated that the first factor on skill discretion contained six items ("I participate with other colleges in decision making", "I participate in implementation of job tasks", "My work develops my abilities", "In my work I have the chance to learn new things", "I have to be creative in my work" and "I undertake different tasks in my work") accounting for 48.7% of the total variance with a Cronbach's alpha of 0.84 and item-total correlations ranging from 0.41 to 0.72. The second factor on decision authority contained three items ("I decide how to perform my job task", "I determine the time and speed of job tasks" and "I solve work problems by myself"). This accounted for 12% of the total variance. Cronbach's alpha was 0.76 and the item-total correlations ranged from 0.44 to 0.53.

#### Job Demands

The domain job demands consisted of a total of 7 items. The Scree plot results identified two factors. Examination of the rotated factor loading showed that one item "I have too many job tasks" loads poorly (<0.5) on both factors, hence it was deleted. The first factor (i.e. time pressure) included three items ("I find trouble to finish my job tasks", "I take regular over times" and "I have limited time to finish my job"). This accounted for 33.7% of the total variance, Cronbach's alpha was 0.71 and the item-total correlations ranged from 0.12 to 0.30. The second factor (i.e. task complexity) held three items ("I work with maximum speed to finish my tasks", "I work under extensive pressure" and "I find my work tasks difficult"). This accounted for 16.1% of the total variance and Cronbach's alpha was 0.53. Item-total correlations ranged from 0.49 to 0.56.

#### Social Support

Twelve items investigated the relationship among co-workers and between workers and supervisors. The Scree plot indicated that two factors were to be retained. The rotated factor loadings indicated that seven items load highly on the first factor on social support ("I find support from supervisors", "I receive positive comments", "My colleagues are helpful", "My supervisors are helpful", "I get personal advice from my colleagues", "My supervisors are considerate" and "I have no contact with other colleagues") accounting for 53.2% of the total variance. Cronbach's alpha was 0.94 and item-total correlations of "social support" ranged from 0.85 to 0.88. The other five items ("The work flow goes smoothly", "I can ask and enquire about my work", "My work tasks depend on other colleagues", "The work atmosphere is comfortable" and "I find support from colleagues") were classified as being related to work flow and accounted for 35.8% of the total variance. Cronbach's alpha was 0.76 and item-total correlations ranged from 0.71 to 0.97.

## Discussion

This is the first study investigating the prevalence of CANS in a population of computer office workers in Sudan. The prevalence of neck and shoulder complaints in the study population was higher than the prevalence of arm, hand and elbow complaints. Since there are no data documenting previous prevalence of CANS in Sudan, the study could not identify whether there is an increase or decrease in the prevalence of CANS. However, a 53% of the study population reporting CANS of at least one week duration over a one-year period is a rather substantial number. This result corresponds with our study among computer workers in the Netherlands with a one-year prevalence of 54% of CANS of at least one week duration [11]. Furthermore, a survey in the Netherlands showed that in 2002 and 2004 28% of the working population reported neck/shoulder or elbow/wrist/hand symptoms in the previous 12 months [4] and that these symptoms were at least partly caused by work.

Figures from developing countries are not abundant. A number of studies in countries such as Indonesia [14] have shown that musculoskeletal disorders are quite prevalent with a proportion of the population affected ranging from 14 to 42%. A Lebanese study, which focused on full-time female homemakers, not involved in the formal labour force and aged between 15 and 59 years, found that 19% had musculoskeletal disorders [15]. Al Wazzan's (2001) study among 204 dentists and dental assistants in Saudi Arabia, showed that 54% of the subjects complained of neck pain [16]. The prevalence found in the Sudanese cohort is comparable to our Dutch data and from other data from the region, thus indicating that CANS is not typical of Western countries.

The majority of the participants in our study were classified as mild cases, while only 9 cases (4%) were classified as severe cases. This is in line with results from other cohort studies. In the already mentioned NUDATA study only 16 of 296 participants with forearm pain met clinical criteria for being a forearm case [8]. In another study, also from Denmark, Andersen and colleagues found that only small proportions (<3%) of participants reported moderate to severe acute and chronic neck and shoulder pain [17]. Based on this pattern of results one might question whether we are dealing with fluctuating daily aches and pain in stead of a health problem which needs serious clinical attention.

Although the majority of subjects in the present study were males, the reported complaints among females were significantly higher. This corresponds with our previous finding in a Dutch cohort study [11] which showed a prevalence of neck symptoms of 24% among men and 42% among women. The earlier mentioned Danish study by Andersen et al showed that women had higher risks of developing neck and shoulder pain [17]. Studies carried out in Lebanon showed a higher prevalence among women than men for all ages for several types of musculoskeletal disorders [15]. These gender differences in the prevalence of musculoskeletal complaints might be explained by differences in exposures to work-related physical and psychosocial risk factors [18].

We have attempted to accurately examine the measurement properties of the Arabic version of the MUEQ. The translation and adaptation of pre-existing questionnaires have two advantages: translated questionnaires provide an efficient way to have a valid and reliable domain that needs to be measured in the targeted language; if the translation shows good psychometric properties, such translated instruments can be used in international comparative studies. However, the assumption is that simple translation is usually successful if the culture of the target population is similar to that of the original population. Because the Sudanese and the Dutch cultures are different, cultural adaptations during translation were essential. The results of the psychometric analyses indicated that the two scales were psychometrically similar [11]. In both questionnaires twelve factors were extracted, explaining approximately 50% of the variance in the Dutch version of the MUEQ compared to 40% in the Arabic version of the MUEQ. Cronbach's alpha coefficients in the Dutch version of the MUEQ ranged from 0.54 to 0.85 compared to 0.48 to 0.94 in the Arabic version. The factors with lower Cronbach's alpha coefficients were in both questionnaires the factors related to computer position and office equipment. In general, cultural differences did not hinder the use of the translated version among the Sudanese cohort. Thus, one can postulate that physical and psychosocial factors related to computer office work are not perceived differently by different cultures. Whether the scales identified by the factor analyses in this study are indeed risk factors for the development of CANS in computer workers, will be the topic of research of a prospective cohort study conducted by our group.

## Conclusion

Data on the prevalence of musculoskeletal disorders have been collected for several decades in Western countries. Studies on the epidemiology of CANS, are mostly restricted to high-income countries, comprising less than 15% of the world population [19]. The current study documents that the prevalence of CANS in computer office workers in Sudan seems to correspond with prevalences of CANS found in other Western and non-Western countries. Furthermore, the study presents a valid and reliable Arabic questionnaire to be used to assess work-related risk factors for the development of CANS. Nevertheless, the psychometric properties of this questionnaire were studied in employees without severe musculoskeletal complaints. Further evaluation of the psychometric properties of the questionnaire studies in other populations may therefore be useful.

## Competing interests

The authors declare that they have no competing interests.

## Authors' contributions

SEand JBS have made substantial contributions to conception and writing. SE did the data analysis and drafted the manuscript. SA has helped in adjusting the study questionnaire and AH has critically revised the manuscript and the statistical analysis. All authors read and approved the final manuscript.

## Supplementary Material

Additional file 1 Appendix 1 the Arabic Upper Extremity Questionnaire (AUEQ). Appendix 1 presents the Arabic Upper Extremity Questionnaire. Click here for file

## References

[B1] Huisstede BM, Miedema HS, Verhagen AP, Koes BW, Verhaar JA (2007). Multidisciplinary consensus on the terminology and classification of complaints of the arm, neck and/or shoulder. Occup Environ Med.

[B2] Bongers PM, Kremer AM, ter Laak J (2002). Are psychosocial factors, risk factors for symptoms and signs of the shoulder, elbow, or hand/wrist?: A review of the epidemiological literature. Am J Ind Med.

[B3] Adedoyin RA, Idowu BO, Adagunodo RE, Owoyomi AA, Idowu PA (2005). Musculoskeletal pain associated with the use of computer systems in Nigeria. Technol Health Care.

[B4] Bongers PM, Ijmker S, Heuvel S van den, Blatter BM (2006). Epidemiology of work related neck and upper limb problems: psychosocial and personal risk factors (part I) and effective interventions from a bio behavioural perspective (part II). J Occup Rehabil.

[B5] Ijmker S, Huysmans M, Blatter BM, Beek AJ van der, van Mechelen W, Bongers PM (2007). Should office workers spend fewer hours at their computer? A systematic review of the literature. Occup Environ Med.

[B6] Andersen JH, Thomsen JF, Overgaard E, Lassen CF, Brandt LP, Vilstrup I, Kryger AI, Mikkelsen S (2003). Computer use and carpal tunnel syndrome: a 1-year follow-up study. Jama.

[B7] Brandt LP, Andersen JH, Lassen CF, Kryger A, Overgaard E, Vilstrup I, Mikkelsen S (2004). Neck and shoulder symptoms and disorders among Danish computer workers. Scand J Work Environ Health.

[B8] Kryger AI, Andersen JH, Lassen CF, Brandt LP, Vilstrup I, Overgaard E, Thomsen JF, Mikkelsen S (2003). Does computer use pose an occupational hazard for forearm pain; from the NUDATA study. Occup Environ Med.

[B9] Lassen CF, Mikkelsen S, Kryger AI, Andersen JH (2005). Risk factors for persistent elbow, forearm and hand pain among computer workers. Scand J Work Environ Health.

[B10] Lassen CF, Mikkelsen S, Kryger AI, Brandt LP, Overgaard E, Thomsen JF, Vilstrup I, Andersen JH (2004). Elbow and wrist/hand symptoms among 6,943 computer operators: a 1-year follow-up study (the NUDATA study). Am J Ind Med.

[B11] Eltayeb S, Staal JB, Kennes J, Lamberts PH, de Bie RA (2007). Prevalence of complaints of arm, neck and shoulder among computer office workers and psychometric evaluation of a risk factor questionnaire. BMC Musculoskelet Disord.

[B12] Streiner DL (2003). Starting at the beginning: an introduction to coefficient alpha and internal consistency. J Pers Assess.

[B13] de Vet HC, Ader HJ, Terwee CB, Pouwer F (2005). Are factor analytical techniques used appropriately in the validation of health status questionnaires? A systematic review on the quality of factor analysis of the SF-36. Qual Life Res.

[B14] Darmawan J (2007). Recommendations from the Community Oriented Program for Control of Rheumatic Disease for data collection for the measurement and monitoring of health in developing countries. Clin Rheumatol.

[B15] Habib RR, Hamdan M, Nuwayhid I, Odaymat F, Campbell OM (2005). Musculoskeletal disorders among full-time homemakers in poor communities. Women Health.

[B16] Al Wazzan KA, Almas K, Al Shethri SE, Al-Qahtani MQ (2001). Back & neck problems among dentists and dental auxiliaries. J Contemp Dent Pract.

[B17] Andersen JH, Harhoff M, Grimstrup S, Vilstrup I, Lassen CF, Brandt LP, Kryger AI, Overgaard E, Hansen KD, Mikkelsen S (2008). Computer mouse use predicts acute pain but not prolonged or chronic pain in the neck and shoulder. Occup Environ Med.

[B18] Hooftman WE, Beek AJ van der, Bongers PM, van Mechelen W (2005). Gender differences in self-reported physical and psychosocial exposures in jobs with both female and male workers. J Occup Environ Med.

[B19] Volinn E (1997). The epidemiology of low back pain in the rest of the world. A review of surveys in low- and middle-income countries. Spine.

