# Supplementary material for: Complaints of the arm, neck and shoulder among computer office workers in Sudan: a prevalence study with validation of an Arabic risk factors questionnaire
Source: Environ Health. 2008 Jun 27;7:33. doi: 10.1186/1476-069X-7-33 (PMC2474607; doi:10.1186/1476-069X-7-33)
Supplement: Additional file 1 — Appendix 1 the Arabic Upper Extremity Questionnaire (AUEQ). Appendix 1 presents the Arabic Upper Extremity Questionnaire. [file 1476-069X-7-33-S1.doc]

**استبيان حول المشاكل في الجهاز العضلي الأعلى** **لدى العاملين والعاملات المستخدمين لأجهزة الكومبيوتر**

**معلومات عامة:**

| 1 | الاسم الثلاثي |  |
| --- | --- | --- |
| 2 | النوع: | ذكر□ أنثى□ |
| 3 | العمر: | 25- 35 □ 36 – 45□ 46 -55□ 65 و ما فوق□ |
| 4 | مكان العمل؟ |  |
| 5 | في أي قسم تعمل؟ |  |
| 6 | كم مدة تعمل في هذا القسم؟ . | ______________ شهر |
| 7 | ما وظيفتك الحالية؟ |  |
| 8 | كم عام أو شهرا تعمل في هذه الوظيفة؟ | ______________ شهر |
| 9 | كم ساعة باليوم تعمل؟ | ________________ ساعة |
| 10 | كم عدد الساعات الاضافية؟ | ___________________ ساعة |
| 11 | كم ساعة باليوم تعمل خلف جهاز الكمبيوتر؟ | _________________ ساعة |
| 12 | كم ساعة باليوم تؤدى مهام أخرى (حضور اجتماعات الخ) | ____________________ساعة |

**وضع العمل مكان العمل**

| 13 | الطاولة التي اعمل فيها ارتفاعها مناسب معي. | نعم □ | لا□ |
| --- | --- | --- | --- |
| 14 | يمكنني التحكم بارتفاع الكرسي. | نعم □ | لا□ |
| 15 | عند استخدامي لجهازالفارة( Mouse)تكون يدي مستندة على الطاولة. | نعم □ | لا□ |
| 16 | كرسي العمل الذي أجلس عليه يسند الجزء الأسفل من ظهرى. | نعم □ | لا□ |
| 17 | جهاز الـ Keyboard يقع أمامي مباشرة. | نعم □ | لا□ |
| 18 | يمكنني الجلوس بشكل أفقي (مستقيم) أمام شاشة الكمبيوتر. | نعم □ | لا□ |
| 19 | بحوزتي حافظة للمستندات تثبت بها المستندات حين طباعتها | نعم □ | لا□ |
| 20 | لدى المساحة الكافية للعمل بمكتبي (طاولتي). | نعم □ | لا□ |

**جلوسك أثناء العمل**

|  |  | دائماً | كثيراً | أحياناً | نادراً | لا يحدث |
| --- | --- | --- | --- | --- | --- | --- |
| 21 | أثناء عملي أجلس مدة طويلة في نفس الوضع. | □ | □ | □ | □ | □ |
| 22 | لأكثر من ساعتين في اليوم تكون أكتافي في وضع مشدود (غير مسترخي). | □ | □ | □ | □ | □ |
| 23 | أثناء عملي أجلس في وضع غير مريح. | □ | □ | □ | □ | □ |
| 24 | عملي يتطلب أداء مهام بها حركات متكررة ( طباعة) | □ | □ | □ | □ | □ |
| 25 | أجد طبيعة عملي مرهقة جسمانياً. | □ | □ | □ | □ | □ |

|  |  | دائماً | كثيراً | أحياناً | نادراً | لا يحدث |
| --- | --- | --- | --- | --- | --- | --- |
| 26 | عندما أطبع يكون ساعدي في خط مستقيم مع أسفل ذراعي (اليد ممتدة) | □ | □ | □ | □ | □ |
| 27 | عندما أعمل يكون رأسي في وضع منحني إلى الأسفل | □ | □ | □ | □ | □ |
| 28 | عندم عندما أعمل يكون رأسي في وضع مائل إلى جهة اليمين أواليسار | □ | □ | □ | □ | □ |
| 29 | عندمأعمل يكون جسدي في وضع مائل إلى جهة اليمين أواليسار | □ | □ | □ | □ | □ |
| 30 | أثناء عملي أجلس بشكل مستقيم ( غير متكىء على جانب) | □ | □ | □ | □ | □ |

**محتوى العمل:**

|  |  | دائماً | كثيراً | أحياناً | نادراً | لا يحدث |
| --- | --- | --- | --- | --- | --- | --- |
| 31 | في وظيفتي يمكنني أن أحدد كيفية إنجاز مهام العمل | □ | □ | □ | □ | □ |
| 32 | 1. عملي يشبع قدراتي | □ | □ | □ | □ | □ |
| 33 | 1. عملي يوفر لي فرص لاكتساب معلومات جديدة | □ | □ | □ | □ | □ |
| 34 | 1. يمكنني أن أشارك في كيفية سير العمل | □ | □ | □ | □ | □ |
| 35 | 1. . أحصل في عملي على مساحة لأتخذ القرار بنفسي | □ | □ | □ | □ | □ |
| 36 | 1. في في عملي يتوجب علىّ أن أكون مبتكرا | □ | □ | □ | □ | □ |
| 37 | 1. . في عملي أحصل على مهام عديدة متنوعة | □ | □ | □ | □ | □ |
| 38 | 1. يمكنني أن أتحكم في تحديد السرعة التي أؤدي بها العمل | □ | □ | □ | □ | □ |
| 39 | 1. أحصل على الفرصة الكافية لحل معضلات العمل بنفسي | □ | □ | □ | □ | □ |

**طبيعة العمل:**

|  |  | دائماً | كثيراً | أحياناً | نادراً | لا يحدث |
| --- | --- | --- | --- | --- | --- | --- |
| 40 | أجد نفسي أعمل تحت ضغط عالٍ | □ | □ | □ | □ | □ |
| 41 | أجد صعوبة في إنجاز مهام عملي في الوقت المحدد | □ | □ | □ | □ | □ |
| 42 | أجد نفسي أعمل ساعات إضافية لانجازمهام عملي في وقتها | □ | □ | □ | □ | □ |
| 43 | 1. أجد مهام عملي صعبة | □ | □ | □ | □ | □ |
| 44 | 1. أجد مهام عملي كثيرة | □ | □ | □ | □ | □ |
| 45 | 1. لا أجد الوقت الكافي في عملي لإنجاز مهامي | □ | □ | □ | □ | □ |
| 46 | 1. أثناء عملي يجب علىّ العمل بسرعة لإنجاز مهامي | □ | □ | □ | □ | □ |

**زمن الراحة (الفسحة)**

|  |  | دائماً | كثيراً | أحياناً | نادراً | لا يحدث |
| --- | --- | --- | --- | --- | --- | --- |
| 47 | أثناء أدائي لعملي لا يكون جسدي في وضع واحد | □ | □ | □ | □ | □ |
| 48 | أثناء عملي أبدل في أدء مهامي | □ | □ | □ | □ | □ |
| 49 | 1. أثناء عملي يمكنني أن أحدد الزمن الذي آخذ فيه راحة أو فسحة | □ | □ | □ | □ | □ |
| 50 | 1. يمكنني بنفسي أن أحدد متى أبدأ في المهمة ومتى أنتهي منها | □ | □ | □ | □ | □ |
| 51 | أثناء عملي اؤدى مهام أخرى لا تتطلب استخدام الكمبيوتر | □ | □ | □ | □ | □ |
| 52 | بعد عملي **لساعتين** متواصلتين خلف الكمبيوتر آخذ راحة لا تقل عن **10** دقائق دقائق | □ | □ | □ | □ | □ |
| 53 | 1. يمكنني أن أقسم مهام عملي إذا رأيت ذلك ضروريا | □ | □ | □ | □ | □ |
| 54 | 1. في اليوم العملي أجد أن زمن فسحة الراحة كافيا | □ | □ | □ | □ | □ |
| 55 | 1. فسحة الافطار أو الشاي تكون خارج المكتب | □ | □ | □ | □ | □ |

**بيئة العمل:**

|  |  | دائماً | كثيراً | أحياناً | نادراً | لا يحدث |
| --- | --- | --- | --- | --- | --- | --- |
| 56 | أجد بيئة عملي جيدة (نظيفة،مضيئة) | □ | □ | □ | □ | □ |
| 57 | داخل المكتب يكون الهواء جاف جداً. | □ | □ | □ | □ | □ |
| 58 | داخل المكتب يكون الهواء حار | □ | □ | □ | □ | □ |
| 59 | أجهزة التبريد تكون باردة جداً | □ | □ | □ | □ | □ |
| 60 | في مكان عملي يتوفر الهواء الطبيعي | □ | □ | □ | □ | □ |
| 61 | بيئة عملي مزعجة ( جلبة ناس) | □ | □ | □ | □ | □ |
| 62 | مكان عملي مضاء بشكل قوي | □ | □ | □ | □ | □ |
| 63 | 1. شاشة الكمبيوتر تعكس إضاءة غرفة المكتب | □ | □ | □ | □ | □ |

**دعم اجتماعي:**

|  |  | دائماً | كثيرً | أحياناً | نادراً | لا يحدث |
| --- | --- | --- | --- | --- | --- | --- |
| 64 | العمل يسير بشكل منظم | □ | □ | □ | □ | □ |
| 65 | يمكنني أن استفسر بشكل كاف في عملي | □ | □ | □ | □ | □ |
| 66 | طبيعة عملي تعتمد كثيراً على زملائي | □ | □ | □ | □ | □ |
| 67 | 1. أصف العلاقات العامة في العمل بأنها مريحة | □ | □ | □ | □ | □ |
| 68 | 1. إذا أخطأت في مهام عملي أجد الدعم والإرشاد الكافي من زملائي | □ | □ | □ | □ | □ |
| 69 | 1. إذا أخطأت في مهام عملي أجد الدعم والإرشاد الكافي من رؤسائي | □ | □ | □ | □ | □ |
| 70 | 1. اشعر بتقييم اجيابى لعملي من زملائي | □ | □ | □ | □ | □ |
| 71 | 1. زملائي في العمل متعاونون | □ | □ | □ | □ | □ |
| 72 | 1. رؤسائي في العمل متعاونون | □ | □ | □ | □ | □ |
| 73 | 1. أجد الدعم النفسي في أموري الخاصة من زملائي | □ | □ | □ | □ | □ |
| 74 | 1. رؤسائي في العمل يقدرون إذا مررت بظروف شخصية صعبة | □ | □ | □ | □ | □ |
| 75 | 1. طبيعة عملي لا توفر لي فرصة التعامل مع زملائي | □ | □ | □ | □ | □ |

**الشكاوى الجسمانية:. الجهاز العضلي الأعلى**

في هذه اللحظة ولمدة **أسبوع** على الأقل أشعر بألم / بشكوى في احد أو أكثر من المناطق الآتية:


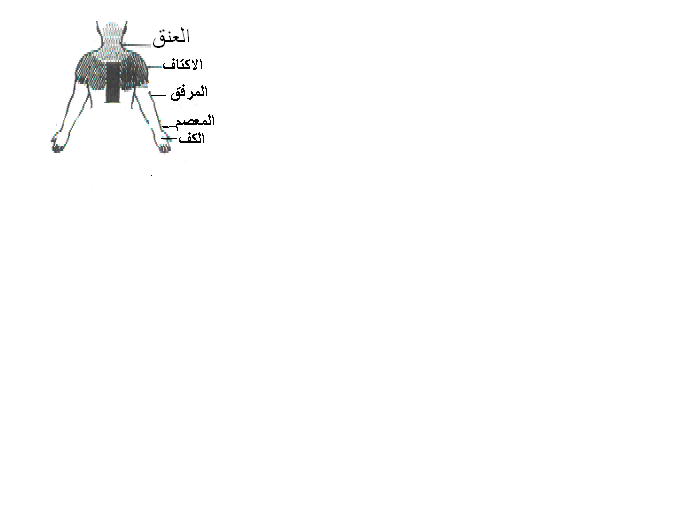


| 76 | **الرقبة العنق** | □ لا  □ نعم |  | |
| --- | --- | --- | --- | --- |
| 77 | **الأكتاف** | □ لا  □ نعم | إذا نعم | □ الأيمن  □ الأيسر  □ الاثنان معاً |
| 78 | 1. **الجزء الأعلى من الذراع** | □ لا  □ نعم | إذا نعم | □ الأيمن  □ الأيسر  □ الاثنان معاً |
| 79 | **المرفق ( الكوع)** | □ لا  □ نعم | إذا نعم | □ الأيمن  □ الأيسر  □ الاثنان معاً |
| 80 | 1. **الجزء الأسفل من الذراع** | □ لا  □ نعم | إذا نعم | □ الأيمن  □ الأيسر  □ الاثنان معاً |
| 81 | 1. **الكف** | □ لا  □ نعم | إذا نعم | □ الأيمن  □ الأيسر  □ الاثنان معاً |
| 82 | 1. **المعصم** | □ لا  □ نعم | إذا نعم | □ الأيمن  □ الأيسر  □ الاثنان معاً |

من هنا يستخدم تعبير( **الجهازالعضلي الأعلى**) من الجسد للتعبيرعن:

**(العنق أو الأكتاف أو الكف أو المعصم أو المرفق أو الذراع**)

| 83 | في العام الماضي شعرث بألم في الجهاز العضلي الأعلى | نعم□ | لا□ |  |
| --- | --- | --- | --- | --- |
| 84 | في العام الماضي أدت شكاوى في الجهاز العضلي الأعلى إلى حد  أنشطتي | نعم□ | لا□ |  |
| 85 | 1. خلال العام الماضي أدت شكاوي في الجهاز العضلي الأعلى إلى 2. الذهاب إلى الطبيب | نعم□ | لا□ |  |
| 86 | 1. تشخيص الطبيب لمشاكلي في الجهاز العضلي الأعلى: | شد عضلي □ | رطوبة □ | أخري حدد |
| 87 | 1. هل ذكر الطبيب سبب للشكوى أعلاه | نعم□ | لا□ | إذا نعم حدد |
| 88 | 1. اُضطررت للتغيب من العمل نتيجة الشكوى من مشاكل الجهاز 2. العضلي الأعلى | نعم□ | لا□ |  |
| 89 | 1. آلام (الجهاز العضلي الأعلى) أدت لفقداني وظيفة من قبل | نعم□ | لا□ |  |
| 90 | 1. أثناء شكوتي من آلام (الجهازالعضلي الأعلى) 2. قلت أنشطتي: في مجال عملي | نعم□ | لا□ |  |
| 91 | 1. أثناء شكوتي من آلام (الجهازالعضلي الأعلى) 2. قلت أنشطتي: في مجالي الاجتماعي | نعم□ | لا□ |  |
| 92 | 1. شكواى الآن من الآم في (الجهازالعضلي الأعلى) نتيجة 2. لحادث/ إصابة | نعم□ | لا□ |  |

الأسئلة القادمة تتعلق ، إذا كنت تشكو في العام الماضي من ألم أو عدم راحة في الجهاز العضلي الأعلى

**(العنق أو الأكتاف أو الكف أو المعصم أو المرفق أو الذراع**)

| 93 | أشعر بألم في الجهاز العضلي الأعلى بعد انتهاء العمل مباشرة | | □ لا  □ نعم | إذا نعم | يزول شعوري الألم بعد قسط من الراحة | □ لا □ نعم |
| --- | --- | --- | --- | --- | --- | --- |
| 94 | أشعر بالإرهاق أو التعب في الجهاز العضلي الأعلى | | □ لا  □ نعم | إذا نعم | ينتهي شعور الإرهاق بعد قسط من الراحة | □ لا □ نعم |
| 95 | أشعر بتقلصات في أصابع يدي. | | □ لا  □ نعم | إذا نعم | تنتهي التقلصات بعد قسط من الراحة | □ لا □ نعم |
| 96 | 1. أشعر بتنميل (خدر) في أصابعي | | □ لا  □ نعم | إذا نعم | ينتهي شعور التنميل بعد قسط من الراحة | □ لا □ نعم |
| 97 | | 1. أشعر بوخزات في أصابعي | □ لا  □ نعم | إذا نعم | يبقى هذا الشعور بعد قسط من الراحة | □ لا □ نعم |
| 98 | | 1. أشعر بضعف في قواي أو ارتخاء في 2. ( الجهاز العضلي الأعلى | □ لا  □ نعم | إذا نعم | يبقى هذا الشعور بعد إنتهاء اليوم العملي | □ لا □ نعم |

| 99 | 1. أعاني من تورم أو انتفاخ في أصابع يدي | □ لا  □ نعم | إذا نعم | يبقى هذا الشعور بعد إنتهاء اليوم العملي | □ لا □ نعم |
| --- | --- | --- | --- | --- | --- |
| 100 | 1. أشعر بتورم/ تقلص/ في( الجهازالعضلي الأعلي). | □ لا  □ نعم |  | |  |
| 101 | 1. أشعر بألم متواصل في (الجهاز العضلي الأعلى) | □ لا  □ نعم |  | |  |
| 102 | 1. لدى الإحساس بتغير في درجة حرارتي أو لون الجلد 2. أو العرق في(الجهاز العضلي الأعلى) | □ لا  □ نعم |  | |  |
| 103 | استخدم سنادة لجهاز الفارة(Mouse ) / سنادة أقدام   1. لتخفيض الضغط على(الجهاز العضلي الأعلى) | □ لا  □ نعم |  | |  |
| 104 | 1. هل تلقيت أي علاج للتخفيف من مشاكلك | □ لا  □ نعم | إذا نعم | | علاج طبيعي □ علاج بلدي البصير□  أدوية مسكنة□  أخرى□ |
| 105 | اشعر إنني في صحة جسمانية جيدة. | □ لا  □ نعم |  | |  |
| 106 | 1. بجانب عملي أودي واجباتي المنزلية بشكل جيد | □ لا  □ نعم |  | |  |
| 107 | 1. استخدم الكمبيوتر في المنزل (للعمل أو الترفيه) | □ لا  □ نعم |  | |  |
